# A qualitative examination of sanctification: Sources and varieties of appraisals of sacredness

**DOI:** 10.1016/j.ijchp.2025.100578

**Published:** 2025-05-02

**Authors:** Elizabeth J. Krumrei-Mancuso, Janet P. Trammell, Jennifer A. Harriger, Joshua A. Evans

**Affiliations:** Pepperdine University, USA

**Keywords:** Sanctification, Spirituality, Religion, Cognitive appraisal, Sacred, Self-transcendence, Emotion

## Abstract

Most mental health professionals are expected to have some basic competency in religious and spiritual issues. Such competency includes knowledge about the role of religion/spirituality in people’s lives, particularly as it relates to mental health. The current research explored people’s cognitive appraisals of sanctification, by which individuals interpret stimuli to be sacred. To gain a better understanding of the spontaneous cognitive appraisals of sanctification people form during daily life, we randomly assigned religious and/or spiritual individuals (*N* = 144) to an event reconstruction of a time they experienced something as sacred, divine, miraculous, spiritual, holy, or blessed. We qualitatively coded 28 features of these event reconstructions. Participants’ most common descriptions involved sanctification of people, relationships, and places that were not considered sacred in and of themselves, but were considered sacred by association to higher powers or transcendent realities. Common features of sacredness reconstructions included emotions and sensory experiences. We also observed differences in the features associated with each sacred adjective (sacred, divine, miraculous, spiritual, holy, or blessed). We discuss implications for mental health professionals and for the advancement of the science of sanctification.

The search for the exotic, the strange, the unusual, the uncommon has often taken the form of pilgrimages, of turning away from the world, the “Journey to the East,” to another country or to a different Religion. The great lesson from the true mystics, from the Zen monks, and now also from the-Humanistic and Transpersonal psychologists-that the sacred is in the ordinary, that it is to be found in one’s daily life, in one’s neighbors, friends, and family, in one’s back yard…

-Abraham H. Maslow (1964/2021, p. viii)

## Introduction

The subjective interpretations individuals form about the stimuli they encounter have powerful implications for their wellbeing. Such interpretations can be referred to as cognitive appraisals, or simply, appraisals. Sanctification is one such appraisal–the cognitive interpretation that an aspect of life is sacred or holy (Mahoney et al., 1999). The discipline of psychology is not equipped to address ontological questions about sacredness, but takes an interest in understanding people’s perceptions of sacredness and the psychological implications thereof. As noted by Maslow (1964/2021), sanctification may once have been thought to relate to explicitly religious destinations and symbols: Mecca, a shrine, a cross, the Star of David, and so forth. But research indicates people can sanctify practically any aspect of life (Pargament et al., 2017). Through sanctification appraisals, people can experience everyday objects, activities, and relationships as having spiritual meaning. Both theists and nontheists can sanctify something by viewing it as having sacred qualities, such as being spiritual, miraculous, holy, or sacred. In addition, theists may view something as a manifestation of a Higher Power, such as believing God or Allah is present in a given object, activity, relationship, or event.

Research suggests sanctification is one underlying mechanism of the link between religiousness/spirituality and beneficial mental and physical health outcomes. A recent meta-analysis (Mahoney et al., 2022) of 63 studies indicated participants endorsed sanctifying a range of activities (e.g., work), relationships (e.g., marriage and parenting), and aspects of self (e.g., the body). The implications of sanctification for people’s lives was noteworthy: greater sanctification was associated with better psychological adjustment and less negative functioning to a small-to-moderate degree.

To date, over 40 published studies have asked participants to indicate the extent to which they characterize some target as possessing sacred qualities, such as ‘holy’, ‘miraculous’, or ‘blessed’. Examined targets have included topics such as marriage, romantic relationships, parenting, adoption, sex, sexuality, forgiveness, one’s body, life, strivings, work, learning, political elections, and therapist-client relationships (see Mahoney et al., 2022). This body of literature has demonstrated that views of sanctification can be a powerful organizing force in people’s lives and that people invest tremendous time and energy into matters they consider sacred (Pargament et al., 2017). Although quantitative scales of sanctification have been used for decades, little qualitative work has examined what natural associations people make to the sacred qualities listed on quantitative measures. Further, little is known about how the sacred qualities frequently used in quantitative research are similar and different from one another in the types of reactions they elicit from participants. In this study, our goal was to examine what people view as sacred in everyday life and whether particular sacred qualities tend to be associated with different sacred objects.

### The current study

A common way of quantitatively assessing people’s perceptions that aspects of life are sacred involves evaluating the extent to which people experience something as possessing sacred qualities, such as being holy or blessed. Our goal was to examine the extent to which people’s spontaneous responses about a time they considered something to possess sacred qualities would reflect established theories about spirituality and self-transcendent experiences (Yaden et al., 2017). For example, we wanted to examine the extent to which people’s spontaneous responses to sacred terms would reflect aspects of a frequently employed definition of sacredness as involving a sacred core and ring (Pargament & Mahoney, 2005). In this pictorial representation of the concept of sacredness, a sacred core contains concepts most central to a person’s conceptualization of sacredness, such as God, the divine, higher powers, or transcendent reality. This is surrounded by a sacred ring, which includes any aspect of life that takes on sacred meaning or value by association with matters in the sacred core. This is the psychological process known as sanctification. We were also interested in the extent to which responses would reflect people’s attempts to get closer to that which they consider sacred, also referred to as a spiritual pathway (the sacred entity being the spiritual destination; Pargament, 2007). Further, we aimed to explore how common it was for people’s memories of a sacred object to reflect a sense of manifestation of God, a form of sanctification that has been examined in the literature. We also aimed to gain a deeper understanding of people’s sanctification of particular domains examined in recent research: the body, physical activity, and nature (Krumrei-Mancuso et al., 2025).

Thus, guided by cognitive appraisal theory, we examined the nuances of sanctification by conducting a qualitative study of event reconstructions of moments of sacredness. We also examined whether features of the reconstructions would differ based on the particular sacred adjectives participants were randomly assigned to recall (e.g., holy, blessed, sacred). The hypotheses, procedures, and analyses were preregistered together with a quantitative companion study athttps://osf.io/8bsxe. All procedures were performed in compliance with relevant laws and institutional guidelines, participants provided informed consent prior to participating, and their privacy rights were observed. This study was approved by Pepperdine University's Institutional Review Board (Protocol #: 21–06–1606; July 6, 2021).

## Method

### Materials

Participants were prompted with: “Spend a few moments thinking about a time you experienced something as [sacred adjective].” One of six sacred adjectives was randomly populated: sacred, divine, miraculous, spiritual, holy, or blessed. Using an event reconstruction method (Grube et al., 2008), participants were asked to describe a time they experienced their assigned adjective with the following writing prompt: “Please describe the time you experienced something as [sacred adjective]. What were the circumstances? What details do you remember? Describe how you felt.” Participants were then presented with an untimed response box.

### Participants

The sample consisted of English-speaking adults from the U.S. (*N* = 144) recruited and paid through the research platform Prolific. Average age was 37.73 years (*SD* = 15.02). In order to be included in the study, participants had to identify as at least slightly spiritual or religious. Within the sample, 61.1 % identified as women, 38.9 % identified as men, and no other gender identifications were selected. Most (90.3 %) participants identified with only one race or ethnicity. Among the sample as a whole, 72.9 % identified as White or European American, 13.2 % as Asian or Asian American, 11.1 % Latinx or Hispanic American, 10.4 % as Black or African American, 4.9 % as American Indian or Alaskan Native, and 3.5 % other. Median household income was 50,000 USD. Regarding highest level of education completed, 1.4 % of the sample had completed some high school, 41.0 % had completed high school, 41.7 % had a bachelor’s degree, 16.0 % had a graduate or professional degree. Regarding spirituality, 38.9 % identified as slightly spiritual, 36.8 % as moderately spiritual, and 24.3 % as very spiritual. In terms of religiousness, 38.2 % of the sample identified as not religious at all, 22.2 % as slightly religious, 27.1 % as moderately religious, and 12.5 % as very religious. In terms of religious activities, 29.1 % of the sample indicated they attended church or other religious meetings a few times per month or more frequently and 55.6 % of the sample indicated they engaged in private religious activities, such as prayer, meditation, or Bible study a few times per month or more frequently. The religions represented included 37.5 % Protestant Christian, 16.7 % Roman Catholic, 0.7 % Mormon, 2.1 % Jewish, 2.1 % Muslim, 0.7 % Hindu, 2.1 % Buddhist, 4.2 % another religion (such as Paganism), and 0.7 % a combination of religions. Among the sample, 20.1 % identified as having no religion, 13.9 % identified as agnostic, and 2.1 % identified as atheist. Regarding political affiliation, 46.5 % identified as democrat, 18.1 % identified as republican, 31.9 % identified as Independent.

### Procedures

#### *Data collection and handling*

The study was administered online via Qualtrics. After completing the informed consent process, participants were assigned using simple randomization to one of six sacred qualities. The adjectives were not defined for participants.

Four participants (from across three of the assigned adjectives) were deleted listwise for choosing not to complete the writing task, indicating they did not know what the sacred adjective meant or had never experienced something associated with the term, or providing a nonsensical response. The number of participants in each condition is displayed in Table 1.

**Table 1:** Qualitative Coding of Phenomena Associated With Sacred Qualities.

Sacredness Code	All cases (*N* = 144)		A. Sacred (*n* = 26)		B. Divine (*n* = 30)		C. Miraculous (*n* = 28)		D. Spiritual (*n* = 19)		E. Holy (*n* = 16)		F. Blessed (*n* = 25)	
	*n*	%	*n*	%	*n*	%	*n*	%	*n*	%	*n*	%	*n*	%
Core	29	20.14	5	19.23	6	20.00	2	7.14	8	42.11	7	43.75	1	4.00
				------		------		---de-		--c--f		--c--f		---de-
Ring	118	81.94	22	84.62	20	66.67	20	71.43	17	89.47	14	87.50	25	100.00
				------		-----f		-----f		------		------		-bc---
*Relation to self and others*														
People and interpersonal relationship	69	47.92	14	53.85	11	36.67	15	53.57	7	36.84	5	31.25	17	68.00
				------		------		------		------		------		------
Self-transcendence	39	27.08	4	15.38	11	36.67	6	21.43	9	47.37	8	50.00	1	4.00
				------		-----f		------		-----f		-----f		-b-de-
*Physicality*														
Sensation	67	46.53	12	46.15	14	46.67	12	42.86	12	63.16	12	75.00	5	20.00
				------		------		------		-----f		-----f		---de-
Physical activity	34	23.61	5	19.23	5	16.67	9	32.14	1	5.26	5	31.25	9	36.00
				------		------		------		------		------		------
Health	23	15.97	0	0.00	1	3.33	8	28.57	3	15.79	0	0.00	11	44.00
				--c--f		--c--f		ab----		------		-----f		ab--e-
Ingestion	18	12.50	1	3.85	16	53.33	1	3.57	0	0.00	0	0.00	0	0.00
				-b----		a-cdef		-b----		-b----		-b----		-b----
Healing	15	10.42	0	0.00	2	6.67	6	21.43	3	15.79	0	0.00	4	16.00
				------		------		------		------		------		------
Physical survival	10	6.94	0	0.00	2	6.67	8	28.57	0	0.00	0	0.00	0	0.00
				--c---		------		a----f		------		------		--c---
Exercise or sport	8	5.56	2	7.69	2	6.67	1	3.57	2	10.53	0	0.00	1	4.00
				------		------		------		------		------		------
Body	0	0.00	0	0.00	0	0.00	0	0.00	0	0.00	0	0.00	0	0.00
				------		------		------		------		------		------
*Location*														
Places	63	43.75	17	65.38	16	53.33	7	.00	9	47.37	9	56.25	5	20.00
				--c--f		------		a-----		------		------		a-----
Travel	40	27.78	15	57.69	12	40.00	4	14.29	3	15.79	5	31.25	1	4.00
				--cd-f		-----f		a-----		a-----		------		ab----
Nature	22	15.28	4	15.38	6	20.00	6	21.43	3	15.79	3	18.75	0	0.00
				------		------		------		------		------		------
*Fortune*														
Luck/gift	54	37.50	1	3.85	4	13.33	23	82.14	4	21.05	1	6.25	21	84.00
				--c--f		--c--f		ab-de-		--c--f		--c--f		ab-de-
Work and earning	12	8.33	0	0.00	0	0.00	4	14.29	0	0.00	0	0.00	8	32.00
				-----f		-----f		------		-----f		------		ab-d--
*Religion*														
Religious	45	31.25	10	38.46	7	23.33	3	10.71	11	57.89	11	68.75	3	12.00
				------		----e-		---de-		--c--f		-bc--f		---de-
Theistic	28	19.44	5	19.23	5	16.67	3	10.71	6	31.58	8	50.00	1	4.00
				------		------		----e-		------		--c--f		----e-
Religious ritual	27	18.75	10	38.46	3	10.00	1	3.57	7	36.84	5	31.25	1	4.00
				--c--f		------		a--d--		--c--f		------		a--d--
Prayer	21	14.58	7	26.92	4	13.33	1	3.57	6	31.58	3	18.75	0	0.00
				-----f		------		---d--		--c--f		------		a--d--
*Spiritual world*														
Supernatural	42	29.17	4	15.38	9	30.00	11	39.29	10	52.63	7	43.75	1	4.00
				---d--		------		-----f		a----f		-----f		--cde-
Spirit/ghost	23	15.97	2	7.69	7	23.33	3	10.71	5	26.32	5	31.25	1	4.00
				------		------		------		------		------		------
Afterlife	6	4.17	1	3.85	1	3.33	0	0.00	2	10.53	1	6.25	1	4.00
				------		------		------		------		------		------
*Lifecycle events*														
Milestone	32	22.22	11	42.31		13.33	5	17.86	2	10.53	3	18.75	7	28.00
				------		------		------		------		------		------
Death	17	11.81	4	15.38	3	10.00	4	14.29	2	10.53	2	12.50	2	8.00
				------		------		------		------		------		------
Birth	11	7.64	0	0.00	1	3.33	3	10.71	0	0.00	1	6.25	6	24.00
				-----f		------		------		------		------		a-----
*Outcome of sanctification*														
Emotion	93	64.58	17	65.38	15	50.00	18	64.29	13	68.42	9	56.25	21	84.00
				------		-----f		------		------		------		-b----

Note. z-scores were used to compare proportions of the presence of sacredness codes between the different sacred qualities. Hyphens and letters indicate pairwise comparisons between adjectives for counts on each sacredness code. A letter indicates two adjectives were different from one another at the *p* < .01 level and a hyphen indicates two adjectives were not different at the *p* < .01 level.

#### *Data coding procedures*

We employed thematic analysis. First, we coded a number of categories established a priori as central to the psychology of religion, including whether the content of what the participant associated with the sacred quality related to: the sacred core or ring associated with Pargament and Mahoney’s definition of sacredness (2005), Yaden et al.’s conceptualization of self-transcendent experience (2017), explicitly religious content (Pargament et al., 2013), and experiencing something as a manifestation of God–which is also known as theistic sanctification (Mahoney et al., 1999). In addition, we decided a priori to code whether the content of what the participant associated with the sacred quality included any of the topics examined by our lab in an associated study (Krumrei-Mancuso et al., 2025): the body, physical activity, or nature. Finally, we coded whether participants described any spiritual pathways for connecting with something sacred (Pargament, 2007) or if the participant described the sacred quality eliciting spiritual emotions (Pargament & Mahoney, 2005). Beyond this deductive approach, we used an inductive approach in which we reviewed responses to discover themes (Braun & Clarke, 2006; Saldaña, 2021). Drawing on both the deductive and inductive approaches, we coded and recoded in an iterative process in which disagreements were discussed between coders until consensus of a final codebook was reached. After finalizing the codebook, one of the authors coded the full dataset, and a new, independent coder who had not been involved in the development of the codebook was provided with training and then coded a random subset of 30 % off the data to evaluate inter-coder reliability.

## Results

### Inter-coder reliability

Each participant's written response was coded for the presence or absence of 28 themes. This resulted in 4032 data points. Primary coding was conducted by one of the authors. A second coder independently coded over 30 % of the dataset, resulting in 1232 pairs of data points. Inter-coder reliability was good: KALPHA with 1000 bootstrap samples = 0.81, 95 % CI [.77, 0.85]. This is above the critical value indicating a reliable rating acceptable for drawing conclusions from the coding (Hayes & Krippendorff, 2007; Krippendorff, 2019).

### Features of sacredness event reconstructions

Table 1 shows the number of times each coded feature was present in the written event reconstructions in total and per assigned sacred quality. Table 2 provides a detailed description of the coded features.

**Table 2:** Features of sacredness event reconstructions.

Sacredness Themes and Codes	Description	Examples
*Conceptualization of sacredness (**Pargament & Mahoney, 2005**;**Pargament* et al.*, 2017*)		
Core	Concepts that lie at the core of the sacred (Pargament & Mahoney, 2005; Pargament et al., 2017)	God, the divine, higher powers, transcendent reality
Ring	A conceptual sacred ring surrounding the sacred core that contains aspect of life that take on divine character or significance based on their association with concepts that lie at the core of the sacred	People, objects, or places that are associated with or represent concepts in the sacred core, such as God or transcendent reality
*Relation to self and others*		
People and interpersonal relationship	A personal relationship or a person with whom the participant has a personal relationship	Family member, loved one, support group
Self-transcendence	Self-transcendent experience (Yaden et al., 2017), involving a temporary mental state characterized by the fading of one’s subjective sense of self as an isolated entity into an experience of unity with other people or one’s surroundings	Sense of oneness or merging with the universe, mystical experience, connecting with past, unseen generations, experiencing awe or elevation, experiencing a cosmic connection to something beyond the self, peak experience, transcendence incited by substance use
*Physicality*		
Sensation	Sensory experience, such as taste, touch, smell, sight, or sound	Hearing music or singing, tasting food, feeling touch
Physical activity, other than exercise or sport	Physical activity involving gross motor movements other than exercise or sport	Walking, sexual activity, physical labor, performing
Health	Physical health and health-promoting activities	Being healthy, having a medical test performed
Ingestion	Eating, drinking, or ingesting drugs	Eating a meal, drinking alcohol, taking drugs
Healing	Physical healing or recovery	Recovering from illness, healing from surgery, recovering from drug addiction
Physical survival	A survival experience	Surviving surgery, a car crash, a hurricane, traumatic child birth, or illness
Exercise or sport	Engaging in exercise or sport	Swimming, deep sea diving, hiking, hunting, running, participating in a race
Body	Participant’s body as the object of sanctification	N/A
*Location*		
Places	Sanctification is connected to a particular place	Chapel, mountains, waterfall, ocean, foreign country, graveyard
*Travel	Journeying to a different location	Travel to a foreign country, travel to a religious site
*Nature	Outdoor, unbuilt environments	Nature park, waterfall, ocean, mountains
*Fortune*		
Luck, gift	Experiencing luck or receiving a gift	Finding an unexpected valuable thing, finding money
Work and earning	Earning money through paid work or similar experiences	Getting a new job, being promoted, selling something
*Religion*		
Religious	Established institutions designed to facilitate spirituality and associated symbols and activities	Religious institutions, places of worship, religious scripture, religious rituals
Theistic	A deity	God, Jesus, Allah
Religious ritual	A spiritual or religious ritual or ceremony	Baptism, wedding, funeral
Prayer	Praying	Prayer to a deity or higher power
*Spiritual world*		
Supernatural	An element that operates outside of the laws of nature	God, spirit, ghost, spiritual world, active ancestors
*Spirit/ghost	Experiences of or connections with a spirit or ghost	Vision or message from a spirit or ghost
*Afterlife	A soul or spirit after human death	Experiencing or sensing a deceased loved one, after-life communication
*Life cycle events*		
Milestone	Physical, spiritual, or relational milestone	Religious confirmation, wedding, recommitment ceremony
Death	Dying and the dead	Death of a loved one, memory of a dead loved one
Birth	Childbirth	Giving birth, holding a baby after birth
*Outcome of sanctification*		
Emotion	Elicited emotion	Love, adoration, gratitude, awe, wonder, joy, happiness, fear, anger, calmness, peace, relief, sadness

⁎ Represents a subcode.

The conceptualization of sacredness as consisting of a sacred core and sacred ring (Pargament & Mahoney, 2005; Pargament et al., 2017) could be supported by the reconstructions. Many more cases described by participants consisted of people, objects, or places that were not considered sacred in and of themselves, but were considered sacred by association to intrinsically sacred higher powers or transcendent realities (82 % of reconstructions referred to content in the sacred ring versus 20 % of reconstructions referred to content in the sacred core). Specific content of the sacred core and ring can be seen in Table 1. Among our U.S. sample, nearly all (95 %) descriptions of the sacred core consisted of theistic references, most commonly God or Jesus. However, in some cultures, an inwardly transcendent sacred core is more commonly observed (Ahmadi & Ahmadi, 2018, 2022). In our reconstructions 27 % of cases involved a self-transcendent experience—a mental state characterized by the fading of one’s subjective sense of self as an isolated entity into an experience of unity with other people or one’s surroundings (Yaden et al., 2017). These descriptions could represent cases of an inwardly transcendent sacred core.

Many of the remaining features listed in Table 1 represent content of the sacred ring, such as living people and interpersonal relationships (48 % of reconstructions); nature (15 % of reconstructions); the pathways people took to grow closer to sources of sacredness (Pargament, 2007), such as religious rituals (19 % of reconstructions) and prayer (15 % of reconstructions); and transformational life experiences, such as healings (10 % of reconstructions) and giving birth (8 % of reconstructions).

As we adopted a definition of spirituality involving connecting to sacredness (Pargament, 1997), the reconstructions, by definition, relate to spirituality. Yet, only a smaller subset (31 %) involved explicitly religious themes, referring to content related to the established institutions designed to facilitate spirituality (Pargament et al., 2013), including religious institutions, places of worship, religious scriptures, and religious rituals such as pilgrimages.

Another common feature was that viewing something as sacred usually elicited memorable emotions (65 % of reconstructions), ranging from spiritual emotions, such as awe and elevation (Pargament & Mahoney, 2005), to basic emotions such as happiness and sadness.

Surprisingly, none of the reconstructions consisted of people sanctifying their bodies, such as making statements that they viewed their bodies as sacred, holy, or miraculous or that they experienced their bodies as a temple of God or experienced God as being present in their bodies. Yet, physical sensations were key to almost half of the reconstructions (47 %), and people sanctified physical activities they could do with their bodies (24 %) as well as their health (16 %).

### Differences in features associated with different sacred qualities

We conducted exploratory *z*-score tests for two population proportions to examine whether each pair of sacred qualities differed from one another in the frequency with which each coded feature was mentioned in the reconstructions. The results are indicated with subscript hyphens and letters below each percentage in Table 1. Given the number of paired comparisons we conducted, we only flagged for consideration any differences significant at *p* < .01.

Participants who were asked to describe something *holy* or *spiritual* were more likely to reference things in the sacred core than participants who were asked to describe some of the other sacred qualities. The term *holy* was more likely to draw out theistic reconstructions than many of the other sacred qualities. On the other hand, the term *blessed* was more likely to elicit descriptions of things in the sacred ring than some of the other sacred adjectives. Particularly, *blessed,* as well as *miraculous,* was more likely to involve references to health, luck, and gifts than many of the other sacred adjectives. *Blessed* was also associated with reconstructions about work and earning. Compared to some other adjectives, *blessed* resulted in more birth stories and descriptions of emotions.

The term *spiritual* was most likely, and the term *blessed* least likely to be associated with supernatural features within the reconstructions. The sacred qualities of *divine, spiritual*, and *holy* were all more likely to elicit memories of self-transcendent experiences than the sacred quality *blessed* was. The sacred qualities of *spiritual* and *holy* were also more likely to result in participants recounting physical sensations as part of the reconstructions than the sacred quality of blessed. The sacred qualities of *spiritual* and *holy* were more likely to draw out religious reconstructions and the sacred qualities of *spiritual* and *sacred* were more likely to result in reconstructions about religious ritual and prayer than some of the other sacred qualities.

The term *sacred* was more likely to elicit reconstructions about places and travel compared to many of the other sacred adjectives. Travel was also more likely to be elicited by the term *divine* than the term *blessed*.

The term *miraculous* stood out as being more likely to elicit reconstructions about physical survival than some of the other sacred qualities. Further, asking participants about a time they experienced something as *divine* was associated with recounting experiences of eating, drinking, or substance use more than any other sacred quality.

## Discussion

The purpose of this study was to understand what people view as sacred in everyday life and to explore whether different sacred qualities are associated with different objects of sanctification. We also sought to consider whether people’s spontaneous responses to sacred adjectives would reflect common theories in the psychology of religion and spirituality.

### What people sanctify in everyday life

Our first goal was to explore what people sanctify in their daily lives. The most common features of our participants' reconstructions of cognitive appraisals of sacred qualities involved the description of emotions, sensations, people and interpersonal relationships, and places. Relatively little attention has been paid to the affective dimension of religious experience (Pargament et al., 2017), though recent work has brought more attention to this topic (Van Cappellen et al., 2023). Common emotional experiences mentioned were happiness, gratitude, awe, grief, fear, and intense feelings of peace and calm. Many also mentioned sensory experiences such as seeing, hearing, or feeling sensations and stimuli perceived as sacred, such as hearing music, spiritual voices, prayers, or experiencing awe invoking sights. Emotions and sensory experiences were often linked in participants’ reconstructions. For example, a participant reported “I remember I felt this new level of calm, and there were these shivers down my spine. It was almost like a trance. I was so calm and relaxed.” Sanctification has previously been associated with better psychological adjustment and less negative functioning (Mahoney et al., 2022). This link might be facilitated by the positive emotions elicited by sanctification, as indicated by many of our participants describing emotions of happiness, gratitude, awe, and peace.

Family and other important interpersonal relationships were also common features of the reconstructions. Previous work has focused on sanctification of important relationships such as marriage, parent-child relationships, and the family system (Fellers et al., 2023; Mahoney et al., 2003). People often view family relationships as sacred and may experience God or spirituality through their participation in such relationships (Mahoney et al., 2003, 2022). In the current study, many participants described important interpersonal relationships as connections to God and the transcendent, detailing how they experienced spirituality and their religious faith in the relational context of their family. In addition, reconstructions about life cycle events and milestones, such as births, deaths, marriages, and baptisms, were often connected to interpersonal relationships.

Participants also often mentioned places in their reconstructions. Places have been identified as aspects of the sacred ring (Pargament & Mahoney, 2005; Pargament et al., 2017). Sometimes the places described were sites of explicit religious significance, such as churches or worship centers, but many sanctification appraisals referred to time spent traveling or in nature. Past studies have examined journeys and pilgrimages, describing the call to the journey, the planning, the journey, and the return as sacred components (Di Giovine & Choe, 2020; Goodnow et al., 2017). Further, the references to nature are consistent with the plethora of research suggesting people experience encounters with sacredness in nature (Naor & Mayseless, 2020).

Our findings support work demonstrating that people can attribute spiritual meaning to difficult or stressful situations (Bélanger-Lévesque et al., 2016). For example, our participants made sanctification appraisals for events in which they, their family, or friends were physically saved from harm, including survival and recovery from illness, accidents, addictions, natural disasters, and successful childbirth.

Participants also provided reconstructions about ongoing spiritual connection with the spirit, soul, or ghosts of deceased loved ones. Our participants’ descriptions of visitations from deceased loved one’s spirits echo previous work noting that ghosts, spiritual presences, and visitations are frequently seen in bereavement narratives (Castelnovo et al., 2015; Kamp et al., 2019; Keen et al., 2013; Kwilecki, 2011; Steffen & Coyle, 2011; Vähäkangas et al., 2022). In our data, reconstructions involving interaction with the afterlife were consistently described in positive terms, suggesting such experiences can provide psychological comfort after the death of a loved one and thereby bolster coping (Vähäkangas et al., 2022),

Finally, we sought to determine whether participants would spontaneously identify their bodies or physical activity in their reconstructions. Interestingly, while some mentioned physical activity as sacred, not one participant mentioned sanctification of his or her own body. One of the most commonly studied targets of sanctification is the body, and body sanctification is associated with a number of positive physical and psychological health outcomes (e.g., Mahoney et al., 2005, 2022). Although our results do not indicate that individuals in our sample do not sanctify their bodies, bodies did not seem to be salient in the minds of participants when asked to describe a recent time in which they experienced something as sacred. The positive outcomes of body sanctification are well documented (e.g., Homan & Boyatzis, 2009; Jacobson et al., 2013, 2016a, 2016b; Kusina & Exline, 2021), but our results indicate people may be more likely to sanctify other aspects of life. Perhaps the greater salience attached to sanctifying interpersonal domains (e.g., family relationships, providing emotional support to loved ones) is the reason they provide more benefits compared to sanctifying noninterpersonal domains such as one’s body (Mahoney et al., 2022).

Taken together, this study reinforces that people can find spiritual meaning in religious and non-religious aspects of life (Pargament et al., 2013). Even aspects of life that seem ordinary can be perceived as sacred (Pargament et al., 2017). Many participants reported that they sanctified emotions, relationships, and places. Sanctification of various aspects of life is associated with positive psychosocial functioning (Mahoney et al., 2022). For example, those who sanctify romantic relationships are less likely to engage in infidelity (Fincham et al., 2010) and greater sanctification of marriage is associated with better communication between spouses (Kusner et al., 2014; Padgett et al., 2019). Similar trends exist in nonmarital relationships (Mahoney et al., 2010). Thus, therapeutic techniques supporting sanctification of interpersonal relationships could prove beneficial in family and couples therapy.

These findings are important for clinicians, as people are more likely to invest energy and derive increased benefits from sanctified dimensions of life (Harriger et al., 2024;Pargament & Mahoney, 2005; Pargament et al., 2017) and also experience greater distress when sanctified aspects are lost or violated (e.g., Krumrei, Mahoney, & Pargament, 2009, Krumrei, Mahoney, & Pargament, 2011. Further, sanctifying loss or difficult life events is a unique way in which individuals might bolster positive coping (Pargament, 2007), thereby offering inspiration or resilience during difficult challenges (Mahoney et al., 2022).

### Exploration of the unique aspects of the sacred adjectives

Our second goal was to explore whether different sacred qualities (e.g., holy, blessed, miraculous, etc.) result in participants recalling particular types of sacred objects. Although the findings are exploratory and therefore would need to be tested in hypothesis-driven future research, some interesting patterns appeared to emerge. First, participants who were asked to describe something *holy, spiritual,* or *blessed* were more likely to reference things in the sacred core and explicitly religious content compared to participants who were asked to describe other sacred qualities, such as attending church, or visiting religious landmarks, such as the Vatican. The term *holy* was also more likely to elicit theistic reconstructions, such as experiencing God, compared to many of the other sacred qualities. Additionally, the targets *spiritual* and *sacred* were more likely to result in reconstructions about religious ritual and prayer, which have been linked to religious growth (Mahoney et al., 2022). Overall, *holy, spiritual, blessed*, and to a lesser extent, *sacred* may be more closely tied to theistic and explicitly religious experiences.

The sacred qualities of *divine, spiritual*, and *holy* also elicited more memories of self-transcendent experiences compared to the sacred quality *blessed*. Self-transcendent experiences involve decreased self-salience and increased feelings of connectedness (Yaden et al., 2017). For example, one participant reported “While praying at church, I just felt one with God.” Beyond connections with a deity, others mentioned feeling connected with the Universe, other believers, or people from the past.

*Blessed*, which was less associated with supernatural features and self-transcendent experiences than other sacred qualities, was more likely to be associated with references to health, luck or gifts, and work or earnings. This is consistent with the definition of a blessing being the favor or protection of a Higher Power. One participant stated “Starting my own business was the ultimate blessing in my life. I was nervous about taking the chance and leaving my regular job, but it turned out to be the best thing I ever did in my life.” Interestingly, some have observed over the past decade that popular culture uses of *blessed* have become a way for people to boast about an accomplishment or incite envy while pretending to be humble (Bennett, 2014). Such contemporary uses of the hashtag *blessed* on social media may be shifting the way the term is used and interpreted in society.

The term *miraculous* was more likely to elicit reconstructions about physical survival than some of the other sacred qualities. Participants described memories about surviving traumatic surgeries, recoveries from illness, or when family or friends were saved from physical harm. This reflects a specific case of how people are likely to make spiritual and religious attributions for their positive life outcomes (DeBono et al., 2020) and how people often find sacred meaning in miracle stories (Korte, 2017). For example, one participant stated “[m]y most miraculous moment was when I, then a heavy drug user and drinker, was literally freed from that bondage the second I accepted Jesus Christ into my heart. People who do not believe in God would find this weird or fake but everyone [saw] the change. It was miraculous. I felt lighter, joy, peace, for the first time since I was a child. It’s something I’ll never forget. And the people who witnessed it [were] just as shocked.”

The term *sacred* was more likely to result in reconstructions containing memories about places and travel compared to many of the other sacred adjectives. This hints at the fact that travel, which serves many social purposes such as vacation and migration, also fulfills sacred purposes when it is part of a pilgrimage or other visit to a sacred location. Past research on sacred travel and pilgrimages align with our findings (Di Giovine & Choe, 2020; Goodnow et al., 2017).

Finally, asking participants about a time they experienced something as *divine* was associated with recounting experiences of eating, drinking, or substance use more than any other sacred quality. Research has examined how food may be a vehicle to a connection with Higher Powers, and consuming food and drink may facilitate movement from the mundane to the sacred (Walden, 2018). In addition, substance use has been recognized as a method for eliciting mysticism and self-transcendent experience (Yaden et al., 2017). However, in our sample, many reconstructions in response to *divine* contained descriptions of food and drinks ranging from cheeseburgers to sushi to wine. One participant wrote about their first time eating at a fancy restaurant and stated, “[e]very single bite was perfection. The ambience, the food, the wine. Everything about that dinner was divine.” This demonstrates how terms with spiritual origins can come to be used symbolically in secular ways. Thus, *divine* can be used to refer to anything ranging from a deity to chocolate.

### Limitations and future directions

This was the first study, to our knowledge, to utilize an event reconstruction method rather than assessing views of sacredness in a global, decontextualized way, however the study was not without limitations. The nature of our sample and our qualitative design limits the generalizability of our findings. We recommend future researchers employ similar methodology with a larger and more diverse group of participants. In addition, we provided participants with sacred adjectives without offering definitions or religious/spiritual cues. This provided an opportunity to examine people’s spontaneous responses to the sacred qualities that are often provided in decontextualized ways on quantitative measures of sanctification. However, this approach can also result in participants interpreting sacred terms in nonspiritual ways, such as using divine to refer to delicious food. This helps illustrate how the sacred qualities used to assess sanctification (e.g., with the Sacred Qualities scales), might also raise thoughts of these terms as they are used in everyday language in secular ways. On the basis of gathering participants’ spontaneous responses to the sacred adjectives in this study, future work might examine how participants respond to these terms when explicitly defined for participants in a religious/spiritual context.

Our findings reiterate that people can view practically any aspect of life through a sacred lens. Our hope is that these qualitative findings give clinicians insight to what this process might look like for clients and that this work advances the science of sanctification by generating new hypotheses with clinical implications.

## Declaration of competing interest

The authors declare that they have no known competing financial interests or personal relationships that could have appeared to influence the work reported in this paper.
